# Characterization of a Secretory YML079-like Cupin Protein That Contributes to *Sclerotinia sclerotiorum* Pathogenicity

**DOI:** 10.3390/microorganisms9122519

**Published:** 2021-12-06

**Authors:** Hongxia Fan, Wenwen Yang, Jiayue Nie, Chen Lin, Jian Wu, Dewei Wu, Youping Wang

**Affiliations:** 1 Key Laboratory of Plant Functional Genomics of the Ministry of Education, Yangzhou University, Yangzhou 225009, China; fanhongxialab407@163.com (H.F.); yww_lab407@163.com (W.Y.); jiayuenie@163.com (J.N.); clinbot@126.com (C.L.); wu_jian@yzu.edu.cn (J.W.); 2 Jiangsu Key Laboratory of Crop Genomics and Molecular Breeding, Yangzhou University, Yangzhou 225009, China

**Keywords:** *Brassica napus*, *Sclerotinia sclerotiorum*, effector, YML079-like cupin protein

## Abstract

*Sclerotinia sclerotiorum* causes devastating diseases in many agriculturally important crops, including oilseed rape and sunflower. However, the mechanisms of *Sclerotinia sclerotiorum* pathogenesis remain poorly understood. In this study, we characterized a YML079-like cupin protein (SsYCP1) from *Sclerotinia sclerotiorum*. We showed that SsYCP1 is strongly expressed and secreted during *Sclerotinia sclerotiorum* infection. *Sclerotinia sclerotiorum* infection was promoted by *SsYCP1* overexpression and inhibited by silencing this gene with synthetic double-stranded RNA. These results collectively indicate SsYCP1 as a putative effector protein that contributes to *Sclerotinia sclerotiorum* pathogenicity. These findings extend our understanding of effector-mediated *Sclerotinia sclerotiorum* pathogenesis and suggest a novel role for YML079-like cupin proteins in plant–pathogen interactions.

## 1. Introduction

*Brassica napus* is an important economic crop, and rapeseed oil is one of the world’s major edible oils. The byproducts of rapeseed, including rapeseed meal and rapeseed stalks, are good resources for animal feed [1,2]. Sclerotinia disease, resulting from infection with *Sclerotinia sclerotiorum*, a necrotrophic ascomycete fungal pathogen, causes significant yield losses and quality reduction in *B. napus* [3,4]. In recent years, many researchers have been trying to develop new *B. napus* cultivars that are resistant to *S. sclerotiorum* through hybrid breeding. However, the progress is relatively slow, largely due to the lack of complete resistant genetic materials in *Brassica* species [3,5,6,7,8,9]. A more comprehensive and in-depth understanding of *S. sclerotiorum* pathogenesis might inspire a new strategy for Sclerotinia disease control.

Effectors play crucial roles in the pathogenesis of a variety of plant pathogens, including viruses, fungi, bacteria, and oomycetes [10]. To date, numerous effectors from various pathogens have been characterized [11,12,13]. These effectors overcome the plant immune system and promote pathogen infection by various mechanisms [10,14,15,16]. Recent studies suggest that, in addition to secreting cell-wall-degrading enzymes and toxins, *S. sclerotiorum* also employs effector proteins to facilitate its infection [17,18]. Cysteine-rich effector SsSSVP1 can interact with QCR8 (a subunit of the mitochondrial respiratory chain cytochrome b-c1 complex) and disturb its subcellular localization to disrupt the function of QCR8 and induce plant cell death [19]. Integrin-like effector SsITL interacts with a chloroplast-localized calcium sensor (CAS) to inhibit salicylic acid accumulation and suppress host immunity during the early stage of infection [20,21]. Cerato-platanin effector SsCP1 interacts with plant-pathogenesis-related protein (PR1) in the apoplast to facilitate *S. sclerotiorum* infection [22]. In spite of this pioneering work, the majority of putative effectors encoded by *S. sclerotiorum*, which could be more than one hundred according to previous bioinformatic predictions [23,24,25], have not been experimentally studied [26,27].

Here, we identified and characterized a novel secretory protein, SsYCP1, from *S. sclerotiorum*. SsYCP1 is a YML079-like cupin protein that is highly expressed and secreted during *S. sclerotiorum* infection. Ectopic overexpression of *SsYCP1* promotes *S. sclerotiorum* infection, while silencing has the opposite effect, suggesting that SsYCP1 is a potential effector protein that contributes to *S. sclerotiorum* pathogenicity. To our knowledge, SsYCP1 is the first YML079-like pathogen cupin protein that can be secreted to function as a potential effector. Interestingly, YML079-like cupin proteins encoded by many other ascomycetes have no secretory signal peptide, indicating that SsYCP1 might be a newly evolved secretory protein to facilitate *S. sclerotiorum* infection.

## 2. Materials and Methods

### 2.1. Bioinformatic Analysis

All homologs of SsYCP1 were searched using the National Center for Biotechnology Information (NCBI) database [28]. The domains of the selected proteins were identified by Pfam (http://pfam.xfam.org, accessed on 18 October 2021) [29]. Signal peptides (SP) were predicted using SignalP-5.0 (http://www.cbs.dtu.dk/services/SignalP, accessed on 18 October 2021) [30], and transmembrane domains were predicted using the TMHMM 2.0 online tool (http://www.cbs.dtu.dk/services/TMHMM, accessed on 18 October 2021). Phylogenetic trees were constructed by neighbor-joining using MEGA-X (iGEM, Boston, MA, USA) [31], and sequence alignments were visualized using Jalview [32].

### 2.2. Yeast Secretion Assay

The pSUC2 vector contains a signal peptide-removed sucrose convertase gene (*SUC2*) [33]. The predicted SP sequences were inserted into the pSUC2-SP vector to fuse with the *SUC2* gene, and the resulting vectors were transformed into the YTK12 yeast strain. Wild-type and transgenic YTK12 yeast strains were spread on YPDA solid medium containing 10 g/L of yeast extract, 20 g/L peptone, 10 g/L glucose, and 20 g/L of agar, or CMD-W medium containing 6.7 g/L yeast nitrogen base, 10 g/L yeast extract, 20 g/L peptone, 10 g/L glucose, and 20 g/L agar, or YPRRA medium containing 10 g/L yeast extract, 20 g/L peptone, 20 g/L cottonseed sugar, and 20 g/L agar. Table S1 lists the primers used for vector construction.

### 2.3. Agrobacteria-Mediated Transient Gene Expression

*SsYCP1* was inserted into the p35S expression vector and then transformed into *Agrobacteria* strain GV3101. Table S1 lists the cloning primer sequences. Cultures of *Agrobacteria* strains were resuspended in 10 mM MES, pH 5.7, containing 10 mM MgCl_2_ and 200 μM acetosyringone, to concentrations of OD_600_ = 0.8. After incubation in the dark at room temperature for two hours, the *Agrobacteria* was injected into *Nicotiana benthamiana* leaves.

### 2.4. Preparation of S. sclerotiorum Mycelium Suspensions

Six *S. sclerotiorum* agar plugs (0.5 cm diameter) were inoculated to 150 mL of potato dextrose broth in a 250 mL conical flask and incubated at 22 °C with shaking at 100 rpm for 24 h. The mycelial balls were collected by filtering through gauze and were washed three times each with ddH_2_O and potato dextrose broth medium. The mycelial balls were then homogenized using a T18 digital ULTRA-TURREX homogenizer (IKA, Staufen, Germany) at 10,000 rpm for 15 min at 4 °C. Ten microliters of OD_600_ = 2.0 suspensions were used to inoculate leaves.

### 2.5. dsRNA Preparation

The Vazyme T7 RNAi Transcription Kit (TR102) was used for dsRNA preparation following the manufacture’s instruction. The target sequences were amplified from cDNA with the primers listed in Table S3 (primers SsYCP1A1 and SsYCP1B1 for amplification of SsYCP1A1, and primers SsYCP1A2 and SsYCP1B2 for SsYCP1A2) as DNA templates for in vitro transcription. Five microliters of dsRNA (900 ng/μL) were mixed with 1 mL of mycelial suspension (OD_600_ = 2.0), and 10 µL was used to inoculate leaves.

### 2.6. Quantitative PCR (qPCR) Analysis

Total RNAs were extracted by Fungal Total RNA Isolation Kit (B518529, Sangon Biotech, Shanghai, China). DNase treatment and first-strand cDNA synthesis were conducted using HiScript 3 RT SuperMix for qPCR (+gDNA wiper) (R323-01, Vazyme Biotech, Nanjing, China). qPCR was performed on StepOnePlus Real-Time PCR System (Thermo, Waltham, MA, USA) using PowerBR Green Master Mixes (Thermo, Waltham, MA, USA). The relative gene expression levels were calculated using the 2^−ΔΔCT^ method [34]. *S. sclerotiorum* Tubulin (MH796665) and *Nicotiana benthamiana* L25 (L18908) were used as internal controls for *S. sclerotiorum* and tobacco genes, respectively.

## 3. Results

### 3.1. SsYCP1 Is Strongly Expressed and Secreted during S. sclerotiorum Infection

A previous study showed that 6% of *S. sclerotiorum* proteins (695 of 11,130) contain SPs and lack predicted transmembrane domains (TM) [23], which could be potential effector proteins. Interestingly, we found that this ratio is significantly (*p* = 0.026, Fisher’s exact test) higher in the cupin superfamily proteins of *S. sclerotiorum*, where 5 out of 24 (17%) cupin proteins meet these criteria (with SP but without TM) (Table S2) [23] (Table S2 and Figure S1). Among these five *S. sclerotiorum* cupins that contain SPs but have no TMs, SS1G_06230 caught our attention, as it belongs to the YML079-like family of the cupin superfamily (also called cupin_5 or DUF985 family), from which no pathogen effector has so far been reported. We renamed SS1G_06230 as SsYCP1 (YML079-like cupin protein 1) for short.

**Figure 1:**
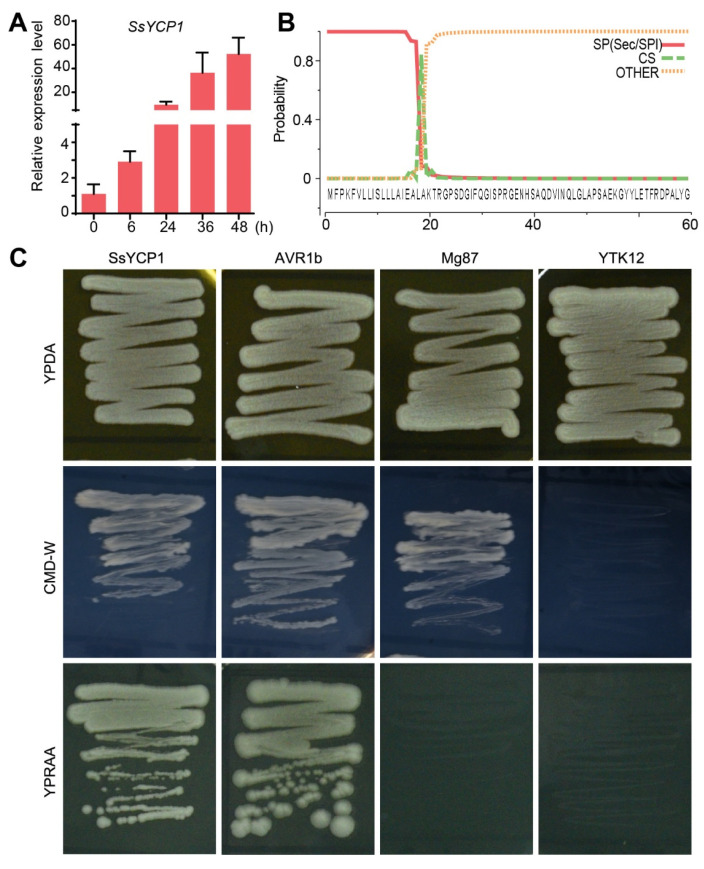
SsYCP1 is strongly expressed and secreted during *S. sclerotiorum* infection. (**A**) Expression of *SsYCP1* at various times after *S. sclerotiorum* inoculation. Leaves were inoculated with mycelial agar plugs and were sampled at 0, 6, 24, 36, and 48 h. Baseline expression of *SsYCP1* (0 h) was set as 1. (**B**) Signal peptide prediction in SsYCP1 protein by SignaIP-5.0. (**C**) Functional verification of the SsYCP1 SP using the YTK12 yeast secretion assay. YPDA (complete medium) was used to verify normal growth, CMD-W medium was used to demonstrate successful transformation of the pSUC2 vector into the yeast strains, and YPRAA medium was used to demonstrate SUC2 secretion. The YTK12 strain was unable to grow on CMD-W and YPRAA media, the YTK12 strain transformed with pSUC2-Mg87 (negative control) was unable to grow on YPRAA medium, and YKT12 strains transformed with pSUC2-SsYCP1 or pSUC2-Avr1b (positive control) grew on all media.

YML079-like family cupin proteins are universally distributed among all life kingdoms (Figure S2). *S. sclerotiorum* encodes three YML079-like cupin proteins. Surprisingly, among these three YML079-like cupins, only SsYCP1 has an SP (Figure S3 and Figure 1B). Moreover, no SPs were identified in the YML079-like proteins of many other ascomycetes, including *Botrytis cinerea*, which is phylogenetically close to *S. sclerotiorum* and encodes only a single YML079-like protein (Table S3), indicating that secretion property of SsYCP1 might be newly evolved by *S. sclerotiorum*. Gene expression analysis showed that SsYCP1 is highly expressed during *S. sclerotiorum* infection, suggesting a role of this gene in *S. sclerotiorum* pathogenesis (Table S2 and Figure 1A).

To investigate whether SsYCP1 is indeed a secretory protein, we carried out the yeast secretion assay. We cloned the SP sequence of *SsYCP1* (*SsYCP1SP*) into the pSUC2 vector to fuse *SsYCP1SP* with the native signal peptide-truncated *SUC2* invertase gene. The pSUC2-SsYCP1SP construct was transformed into the yeast strain YTK12, which cannot survive on YPRAA medium due to the lack of endogenous invertase. The YTK12 strains transformed with pSUC2-SsYCP1SP, as well as with pSUC2-AVR1b, which contains the SP sequence of the *Phytophthora sojae* effector *AVR1b* (positive control), were able to grow on YPRAA medium, suggesting that the SP of SsYCP1 (as well as the SP of AVR1b) allows the secretion of SUC2 invertase [33]. By contrast, the YTK12 strain transformed with *SUC2* fused with the N-terminal sequence of *Magnaporthe* *oryzae Mg87* (which has no secretion ability and served as the negative control), or the original YTK12 strain, did not survive on YPRAA medium [35]. These results demonstrate that the SP of SsYCP1 is functional.

### 3.2. SsYCP1 Overexpression Promotes S. sclerotiorum Infection

**Figure 2:**
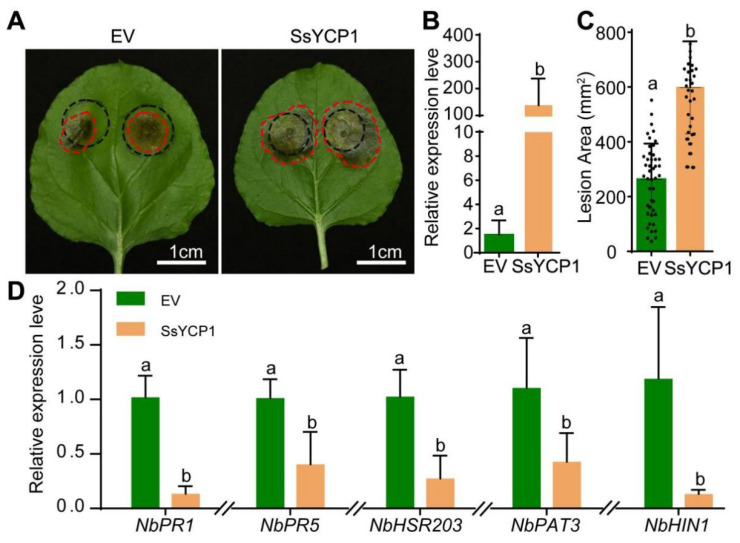
*S. sclerotiorum* infection is promoted by SsYCP1 overexpression. (**A**) Tobacco leaves expressing *SsYCP1* were more susceptible to *S. sclerotiorum*. Leaves were infiltrated with *Agrobacteria* containing the p35S empty vector (EV) or p35S-SsYCP1 (SsYCP1) as indicated, and *S. sclerotiorum* was inoculated 48 h after agroinfiltration. Leaves were photographed after 48 h; representative images are shown. Black circles indicate the leaf area infiltrated with *Agrobacteria*, and red circles indicate *S. sclerotiorum*-induced infections. (**B**) The expression of *SsYCP1* was significantly higher in tobacco leaves agroinfiltrated with p35S-SsYCP1 (SsYCP1) than in the control leaves (EV). Leaves were injected with *S. sclerotiorum* 48 h after agroinfiltration and analyzed by qPCR after 24 h. Data are means ± SD; *n* = 3; Different lowercase letters means *p* < 0.05 (one-way ANOVA followed by Duncan’s test). (**C**) Quantification of *S. sclerotiorum*-induced lesions shown in (**A**), measured by ImageJ. Data are means ± SD; *n* = 34; Different lowercase letters means *p* < 0.05 (one-way ANOVA followed by Duncan’s test). (**D**) The expression of plant defense-related genes was suppressed by SsYCP1. Leaves were inoculated with *S. sclerotiorum* 48 h after agroinfiltration with p35S-SsYCP1 (SsYCP1) or p35S empty vector (EV), and gene expression was analyzed by qPCR 24 h after *S. sclerotiorum* inoculation. Data are means ± SD; *n* = 3; Different lowercase letters means *p* < 0.05 (one-way ANOVA followed by Duncan’s test).

We investigated the effects of SsYCP1 overexpression on *S. sclerotiorum* pathogenicity. We inserted *SsYCP1* without its SP into the p35S vector, where the expression of *SsYCP1* is controlled by the constitutive CaMV 35S promoter. The resulting p35S-SsYCP1 construct was transformed into Agrobacteria and ectopically expressed in tobacco leaves by agroinfiltration. We then inoculated *S. sclerotiorum* onto the tobacco leaves 48 h after agroinfiltration. Increased expression of *SsYCP1* in these leaves in comparison with leaves agroinfiltrated with the empty vector was confirmed by qPCR (Figure 2B). Furthermore, as shown in Figure 2C, *S. sclerotiorum* caused significantly larger lesions on the tobacco leaves overexpressing *SsYCP1* than on control leaves, suggesting that ectopic overexpression of *SsYCP1* can promote *S. sclerotiorum* infection. Consistent with this observation, we found that the expressions of plant-defense-related genes, such as *PR1*, *PR5*, and *HSR203*, were inhibited by *SsYCP1* overexpression (Figure 2D).

### 3.3. Silencing of SsYCP1 Inhibits S. sclerotiorum Infection

**Figure 3:**
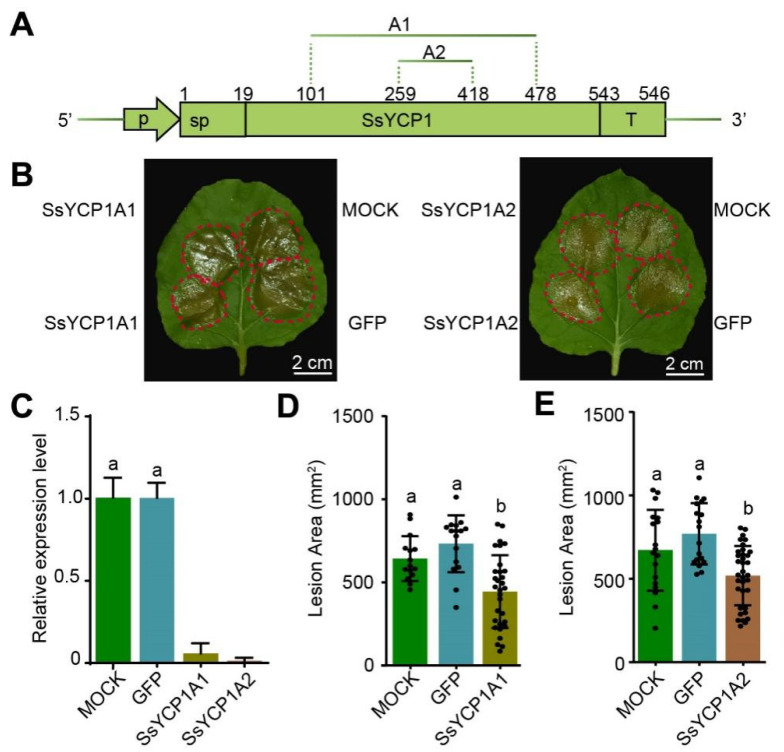
Silencing SsYCP1 reduces *S. sclerotiorum* pathogenicity. (**A**) Schematic diagram of *SsYCP1* dsRNA-targeting regions. p: promoter, sp: signal peptide, A1 (101–478 bp), A2 (259–418 bp) represent two dsRNA, t: terminator, 5′: 5′UTR, 3′: 3′UTR. (**B**) *S. sclerotiorum* caused smaller lesions when *SsYCP1* was silenced by dsRNA. dsRNA (SsYCP1A1 and SsYCP1A2) were transcribed in vitro and mixed with *S. sclerotiorum* mycelium suspensions for inoculation. *S. sclerotiorum* mycelium suspensions mixed with equal volumes of RNase-free ddH_2_O (mock) or with equal concentrations of dsRNA-targeting GFP (GFP) were used as negative controls. Leaves were photographed after 48 h and representative images are shown. (**C**) Expression of *SsYCP1* was knocked down by dsRNA. The expression of *SsYCP1* in leaves of Figure (**B**) was examined by qPCR. Data are means ± SD; *n* = 3; Different lowercase letters means *p* < 0.05 (one-way ANOVA followed by Duncan’s test). (**D**,**E**) Quantification *S. sclerotiorum*-induced lesions on leaves of (**B**). Lesion sizes were measured by ImageJ. Data are means ± SD; *n* = 20; Different lowercase letters means *p* < 0.05 (one-way ANOVA followed by Duncan’s test).

To confirm the positive role of SsYCP1 in *S. sclerotiorum* pathogenesis, we performed the RNAi experiments using synthesized double-strand RNA (dsRNA) that target to *SsYCP1*. We designed two dsRNA, SsYCP1A1 and SsYCP1A2 (Figure 3A). siFi21 software analysis showed that SsYCP1A1 and SsYCP1A2 can specifically target SsYCP1 without any predictable off-target sites (Figure S4).

SsYCP1A1, SsYCP1A2, and GFP-dsRNA (dsRNA targeting *GFP*) were synthesized by in vitro transcription and were then inoculated onto tobacco leaves together with *S. sclerotiorum. S. sclerotiorum* premixed with dsRNA targeting *GFP* or an equal volume of water served as controls. *SSYCP1* expression was monitored by qPCR, which showed significantly reduced expression of *SSYCP1* in SsYCP1A1- or SsYCP1A2-inocuated leaves in comparison with the controls (Figure 3C), confirming the effective silencing of *SsYCP1*. Furthermore, the *S. sclerotiorum*-caused lesion sizes on leaves co-inoculated with SsYCP1A1 or SsYCP1A2 were reduced compared to the leaves co-inoculated with GFP-dsRNA or water (Figure 3B–E), indicating that *SsYCP1* silencing represses *S. sclerotiorum* infection. Consistent with Figure 2, these results verify that SsYCP1 promotes *S. sclerotiorum* infection.

## 4. Discussion

*S. sclerotiorum*, a typical necrotrophic fungus with broad host range, is one of the most devastative plant pathogens, causing huge economic loss worldwide [36]. A lack of knowledge of *S. sclerotiorum* pathogenicity hinders efforts to develop effective methods to control it [17,37]. Although *S. sclerotiorum* encodes many putative effector proteins, only a few of them have been studied [23,24,25,26,27]. Here, we characterized a novel new protein, SsYCP1, in *S. sclerotiorum*. SsYCP1 was found to be strongly expressed and secreted during infection and was positively correlated with *S. sclerotiorum* pathogenicity, suggesting that SsYCP1 is a putative effector protein that contributes to *S. sclerotiorum* pathogenesis (Figure 1C, Figure 2 and Figure 3).

SsYCP1 is a YML079-like cupin protein (Table S1 and Figure S2). Our study presents the first example of YML079-like pathogen cupin proteins that can be secreted to function as a putative effector and promote pathogen infection (Figure 1C, Figure 2 and Figure 3). It is noteworthy that, unlike *S. sclerotiorum*, many other ascomycete fungi, including *Botrytis cinerea* and *Aspergillus oryzae*, encode only one YML079-like cupin protein that lacks a secretory signal peptide (Table S3), indicating that the secretion property of SsYCP1 might be newly evolved by *S. sclerotiorum*. It would be interesting to experimentally investigate whether YML079-like proteins encoded by other pathogens are secretory.

Cupins are a superfamily of topologically conserved but functionally diversified proteins that exist in all kingdoms of life [38]. The reported functions of cupins include isomerases, epimerase, dioxygenase, and nonenzymatic storage proteins [39,40,41,42,43]. The cupin superfamily is classified into 69 families in the Pfam database, and the YML079-like family is one of them [29]. The crystal structure of the YML079w protein from *Saccharomyces cerevisiae* revealed that YML079-like family cupin proteins may adopt a conserved jelly-roll fold [44]. However, the biological functions of the YML079-like cupins remain largely unknown [45]. Previous studies have shown that a YML079-like protein (BbDUF985) from *Branchiostoma belcheri* may function as a phospho-glucose isomerase in the metabolism of nucleotides [46,47]. In this study, we showed that the YML079-like protein SsYCP1 from *S. sclerotiorum* functions as a putative effector to promote the pathogen infection, which represents the first example of such a protein in the YML079-like family. Interestingly, some fungal proteins from other cupin families have also been reported to contain secretory peptides and potentially function as effectors [48,49], indicating a possible important role of cupin superfamily proteins in the pathogenesis of fungal pathogens. Future studies are needed to further reveal how SsYCP1 promotes *S. sclerotiorum* infection, which might not only shed light on the biological functions of YML079-like cupin proteins but also uncover novel mechanisms underlying plant–pathogen interactions.

## 5. Conclusions

In this study, we characterized a novel YML079-like cupin superfamily protein SsYCP1 from *S. sclerotiorum*. SsYCP1 has a functional secretory SP and is highly expressed during *S. sclerotiorum* infection. Ectopic overexpression of *SsYCP1* in plants promoted *S. sclerotiorum* infection, whereas *SsYCP1* silencing by synthetic double-stranded RNA suppressed *S. sclerotiorum* infection, demonstrating a positive role of SsYCP1 in *S. sclerotiorum* pathogenesis. SsYCP1 is the first YML079-like cupin protein that can act as a putative effector to promote pathogen infection. Moreover, many YML079-like cupin proteins encoded by many other ascomycete fungi appear to have no secretion property, suggesting that SsYCP1 is a secretory protein newly evolved by *S. sclerotiorum* to promote its infection. Our work provides new insights into effector-mediated pathogenesis of *S. sclerotiorum* and highlights the potentially important role of YML079-like cupin proteins in the interactions between plants and pathogens.

## Data Availability

Not applicable.

## Supplementary Materials

The following are available online at https://www.mdpi.com/article/10.3390/microorganisms9122519/s1, Figure S1: Cupin proteins of *S. sclerotiorum* are rich in secretory proteins, Figure S2: The taxonomic distribution of YML079-like cupin proteins, Figure S3: Sequence alignment of the three YML079-like family cupin proteins in *S. sclerotiorum*, Figure S4: Off-target site prediction for the two SsYCP1 dsRNA (SsYCP1A1 and SsYCP1A2), Table S1: List of primers used in this study, Table S2: All cupin domain-containing proteins in *S. sclerotiorum*, Table S3: Secretory signal peptide analysis of the 188 YML079-like cupin proteins in ascomycetes.

## References

[1] Zubair M., Maqbool F., Mehmood I., Muzammil S., Waseem M., Imran M., Nadeem H.U., Azeem F., Siddique M.H. (2021). Rapeseed oil. Green Sustainable Process for Chemical and Environmental Engineering and Science.

[2] Friedt W., Tu J., Fu T. (2018). Academic and economic importance of Brassica napus rapeseed. The Brassica Napus Genome.

[3] Neik T., Barbetti M., Batley J. (2017). *Brassica napus* current status and challenges in identifying disease resistance genes in *Brassica napus*. Front. Plant Sci..

[4] Sharma P., Meena P., Verma P., Saharan G., Mehta N., Singh D., Kumar A. (2016). *Sclerotinia sclerotiorum* (Lib) de Bary causing Sclerotinia rot in oilseed Brassicas: A review. J. Oilseed Brassica.

[5] Falak I., McNabb W., Hacault K., Patel J. Field performance of Brassica napus L. spring canola hybrids with improved resistance to Sclerotinia stem rot. Proceedings of the International Rapeseed Congress.

[6] Han-zhong W., Gui-hua L., Yuan-ben Z., Xin-fa W., Qing Y. (2003). Breeding of a Brassica napus cultivar Zhongshuang No. 9 with high-resistance to *Sclerotinia sclerotiorum* and dynamics of its important defense enzyme activity. J. Integr. Agric..

[7] Wang Z., Zhang W.-H., Ma L.-Y., Li X., Tan X.-L. (2020). Overexpression of Brassica napus NPR1 enhances resistance to *Sclerotinia sclerotiorum* in oilseed rape. Physiol. Mol. Plant Pathol..

[8] Wang Z., Wan L., Xin Q., Chen Y., Zhang X., Dong F., Hong D., Yang G. (2018). Overexpression of OsPGIP2 confers *Sclerotinia sclerotiorum* resistance in Brassica napus through increased activation of defense mechanisms. J. Exp. Bot..

[9] Derbyshire M.C., Denton-Giles M. (2016). The control of sclerotinia stem rot on oilseed rape (Brassica napus): Current practices and future opportunities. Plant Pathol..

[10] Hogenhout S.A., Van der Hoorn R.A., Terauchi R., Kamoun S. (2009). Emerging concepts in effector biology of plant-associated organisms. Mol. Plant-Microbe Interact..

[11] Selin C., de Kievit T.R., Belmonte M.F., Fernando W. (2016). Elucidating the role of effectors in plant-fungal interactions: Progress and challenges. Front. Microbiol..

[12] Deslandes L., Rivas S. (2012). Catch me if you can: Bacterial effectors and plant targets. Trends Plant Sci..

[13] Wang Y., Tyler B.M., Wang Y. (2019). Defense and counterdefense during plant-pathogenic oomycete infection. Annu. Rev. Microbiol..

[14] Franceschetti M., Maqbool A., Jiménez-Dalmaroni M.J., Pennington H.G., Kamoun S., Banfield M.J. (2017). Effectors of filamentous plant pathogens: Commonalities amid diversity. Microbiol. Mol. Biol. Rev..

[15] Toruño T.Y., Stergiopoulos I., Coaker G. (2016). Plant-pathogen effectors: Cellular probes interfering with plant defenses in spatial and temporal manners. Annu. Rev. Phytopathol..

[16] Friesen T., Faris J. (2021). Characterization of effector-target interactions in necrotrophic pathosystems reveals trends and variation in host manipulation. Annu. Rev. Phytopathol..

[17] Xu L., Li G., Jiang D., Chen W. (2018). *Sclerotinia sclerotiorum*: An evaluation of virulence theories. Annu. Rev. Phytopathol..

[18] Kabbage M., Yarden O., Dickman M.B. (2015). Pathogenic attributes of *Sclerotinia sclerotiorum*: Switching from a biotrophic to necrotrophic lifestyle. Plant Sci..

[19] Lyu X., Shen C., Fu Y., Xie J., Jiang D., Li G., Cheng J. (2016). A small secreted virulence-related protein is essential for the necrotrophic interactions of *Sclerotinia sclerotiorum* with its host plants. PLoS Pathog..

[20] Zhu W., Wei W., Fu Y., Cheng J., Xie J., Li G., Yi X., Kang Z., Dickman M.B., Jiang D. (2013). A secretory protein of necrotrophic fungus *Sclerotinia sclerotiorum* that suppresses host resistance. PLoS ONE.

[21] Tang L., Yang G., Ma M., Liu X., Li B., Xie J., Fu Y., Chen T., Yu Y., Chen W. (2020). An effector of a necrotrophic fungal pathogen targets the calcium-sensing receptor in chloroplasts to inhibit host resistance. Mol. Plant Pathol..

[22] Yang G., Tang L., Gong Y., Xie J., Fu Y., Jiang D., Li G., Collinge D., Chen W., Cheng J. (2018). A cerato-platanin protein SsCP1 targets plant PR1 and contributes to virulence of *Sclerotinia sclerotiorum*. New Phytol..

[23] Derbyshire M., Dentongiles M., Hegedus D., Seifbarghy S., Rollins J., Kan J.V., Seidl M.F., Faino L., Mbengue M., Navaud O. (2017). The complete genome sequence of the phytopathogenic fungus *Sclerotinia sclerotiorum* reveals insights into the genome architecture of broad host range pathogens. Genome Biol. Evol..

[24] Guyon K., Balagué C., Roby D., Raffaele S. (2014). Secretome analysis reveals effector candidates associated with broad host range necrotrophy in the fungal plant pathogen *Sclerotinia sclerotiorum*. BMC Genom..

[25] Heard S., Brown N.A., Hammond-Kosack K. (2015). An interspecies comparative analysis of the predicted secretomes of the necrotrophic plant pathogens *Sclerotinia sclerotiorum* and Botrytis cinerea. PLoS ONE.

[26] Seifbarghi S., Borhan M.H., Wei Y., Ma L., Coutu C., Bekkaoui D., Hegedus D.D. (2020). Receptor-Like Kinases BAK1 and SOBIR1 are required for necrotizing activity of a novel group of *Sclerotinia sclerotiorum* necrosis-inducing effectors. Front. Plant Sci..

[27] Xia S., Xu Y., Hoy R., Zhang J., Qin L., Li X. (2019). *Sclerotinia sclerotiorum* the notorious soilborne pathogenic fungus: An update on genes studied with mutant analysis. Pathogens.

[28] Klimke W., Agarwala R., Badretdin A., Chetvernin S., Ciufo S., Fedorov B., Tatusova T. (2009). The national center for biotechnology information’s protein clusters database. Nucleic Acids Res..

[29] Finn R.D., Bateman A., Clements J., Coggill P., Eberhardt R.Y., Eddy S.R., Heger A., Hetherington K., Holm L., Mistry J. (2014). Pfam: The protein families database. Nucleic Acids Res..

[30] Nielsen H. (2017). Predicting secretory proteins with SignalP. Protein Function Prediction.

[31] Kumar S., Stecher G., Li M., Knyaz C., Tamura K. (2018). MEGA X: Molecular evolutionary genetics analysis across computing platforms. Mol. Biol. Evol..

[32] Waterhouse A.M., Procter J.B., Martin D.M., Clamp M., Barton G.J. (2009). Jalview Version 2-a multiple sequence alignment editor and analysis workbench. Bioinformatics.

[33] Dou D., Kale S.D., Wang X., Jiang R.H., Bruce N.A., Arredondo F.D., Zhang X., Tyler B.M. (2008). RXLR-mediated entry of *Phytophthora sojae* effector Avr1b into soybean cells does not require pathogen-encoded machinery. Plant Cell.

[34] Livak K.J., Schmittgen T.D. (2001). Analysis ofrelative gene expression data using real-time quantitative PCR and the 2^−ΔΔCT^ method. Methods.

[35] Gu B., Kale S.D., Wang Q., Wang D., Pan Q., Cao H., Meng Y., Kang Z., Tyler B.M., Shan W. (2011). Rust secreted protein Ps87 is conserved in diverse fungal pathogens and contains a RXLR-like motif sufficient for translocation into plant cells. PLoS ONE.

[36] Liang X., Rollins J. (2018). Mechanisms of broad host range necrotrophic pathogenesis in *Sclerotinia sclerotiorum*. Phytopathology.

[37] O’Sullivan C.A., Belt K., Thatcher L.F. (2021). Tackling Control of a Cosmopolitan Phytopathogen: Sclerotinia. Front. Plant Sci..

[38] Khuri S., Bakker F.T., Dunwell J.M. (2001). Phylogeny, function, and evolution of the cupins, a structurally conserved, functionally diverse superfamily of proteins. Mol. Biol. Evol..

[39] El Hadrami A., Islam M., Adam L.R., Daayf F. (2015). A cupin domain-containing protein with a quercetinase activity (VdQase) regulates Verticillium dahliae’s pathogenicity and contributes to counteracting host defenses. Front. Plant Sci..

[40] Giraud M.-F., Leonard G.A., Field R.A., Berlind C., Naismith J.H. (2000). RmlC, the third enzyme of dTDP-L-rhamnose pathway, is a new class of epimerase. Nat. Struct. Biol..

[41] Uberto R., Moomaw E.W. (2013). Protein similarity networks reveal relationships among sequence, structure, and function within the cupin superfamily. PLoS ONE.

[42] Agarwal G., Rajavel M., Gopal B., Srinivasan N. (2009). Structure-based phylogeny as a diagnostic for functional characterization of proteins with a cupin fold. PLoS ONE.

[43] Andreeva A., Howorth D., Brenner S.E., Hubbard T.J., Chothia C., Murzin A.G. (2004). SCOP database in 2004: Refinements integrate structure and sequence family data. Nucleic Acids Res..

[44] Zhou C.Z., Meyer P., Cheruel S.q., De La Sierra Gallay I.L., Collinet B., Graille M., Van Tilbeurgh H. (2005). Crystal structure of the YML079w protein from *Saccharomyces cerevisiae* reveals a new sequence family of the jelly-roll fold. Protein Sci..

[45] Gough J., Karplus K., Hughey R., Chothia C. (2001). Assignment of homology to genome sequences using a library of hidden Markov models that represent all proteins of known structure. J. Mol. Biol..

[46] Gaowa S., Zhang S. (2009). Identification, expression, function and localization of a DUF985 domain-containing hypothetical gene from amphioxus Branchiostoma belcheri. Comp. Biochem. Physiol. Part B Biochem. Mol. Biol..

[47] Du Y., He Y.-X., Gaowa S., Zhang X., Chen Y., Zhang S.-C., Zhou C.-Z. (2010). Crystal structures of the apo and GDP-bound forms of a cupin-like protein BbDUF985 from Branchiostoma belcheri tsingtauense. Proteins-Struct. Funct. Bioinform..

[48] Prasanth C.N., Viswanathan R., Malathi P., Sundar A.R. (2019). Comparative transcriptome analysis of candidate secretory effector proteins from *Colletotrichum falcatum* infecting sugarcane. Agri Gene.

[49] Lopez D., Ribeiro S., Label P., Fumanal B., Venisse J.-S., Kohler A., De Oliveira R.R., Labutti K., Lipzen A., Lail K. (2018). Genome-wide analysis of *Corynespora cassiicola* leaf fall disease putative effectors. Front. Microbiol..

