# Supplementary material for: Characterization of a Secretory YML079-like Cupin Protein That Contributes to Sclerotinia sclerotiorum Pathogenicity
Source: Microorganisms. 2021 Dec 6;9(12):2519. doi: 10.3390/microorganisms9122519 (PMC8704077; doi:10.3390/microorganisms9122519)
Supplement: Supplementary file 1 [file microorganisms-09-02519-s001.zip › microorganisms-1475664-supplementary.pdf]

Supplementary Materials

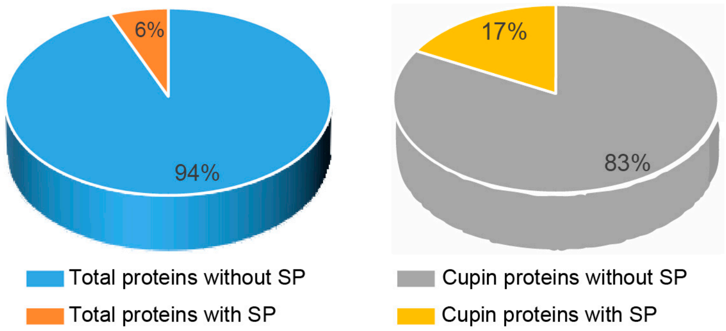

Figure S1. Cupin proteins of *S. sclerotiorum* are rich in secretory proteins

The total number of proteins encoded by *S. sclerotiorum* is approximately 11,130, which contains about 695 proteins that contain predicted secretory signal peptides (SP) but lack predicted transmembrane domains [1]. The total number of cupin domain-containing proteins encoded by *S. sclerotiorum* is 24 as analyzed using the SUPERFAMILY website (https://supfam.mrc-lmb.cam.ac.uk/SUPERFAMILY/)[2], among which 5 cupin proteins have SPs and lack predicted transmembrane domains as predicted by SignalP-5.0 and TMHMM 2.0.

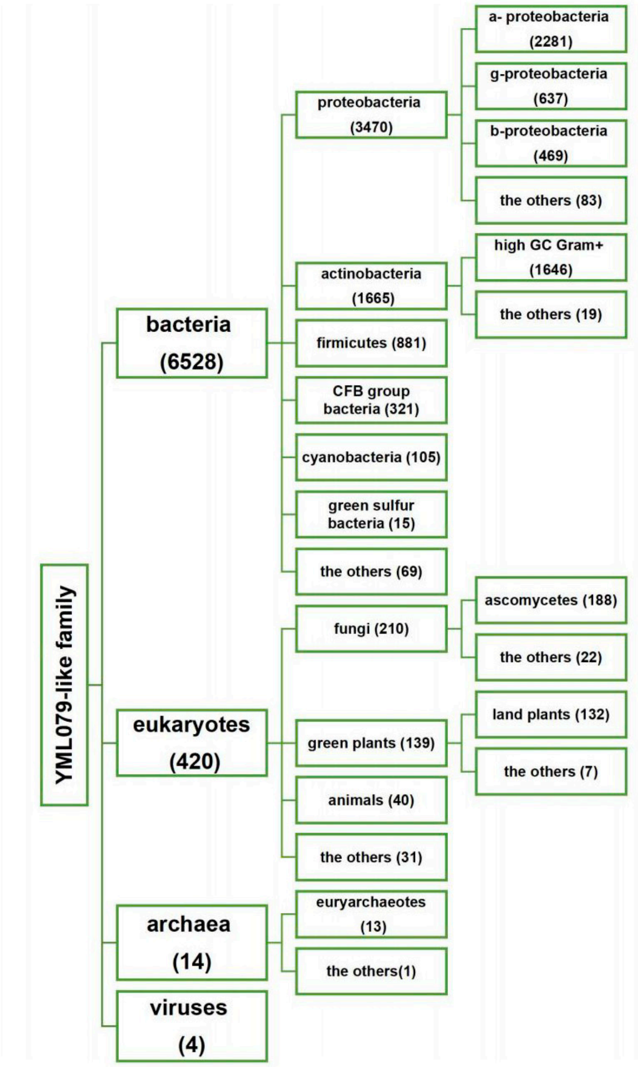

Figure S2. The taxonomic distribution of YML079-like cupin proteins

The taxonomic distribution of all annotated YML079-like family cupin proteins (also named as DUF985 domain containing proteins in NCBI database) was analyzed using NCBI.

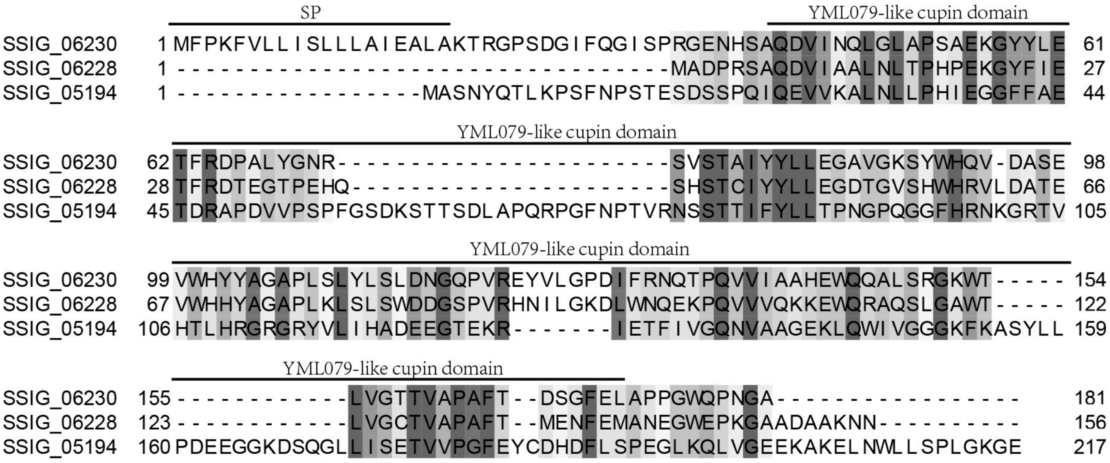

Figure S3. Sequence alignment of the three YML079-like family cupin proteins in *S. sclerotiorum*

The sequences of the three YML079-like family cupins in *S. sclerotiorum* were aligned using MEGA-X. The alignment result was visualized using Jalview.

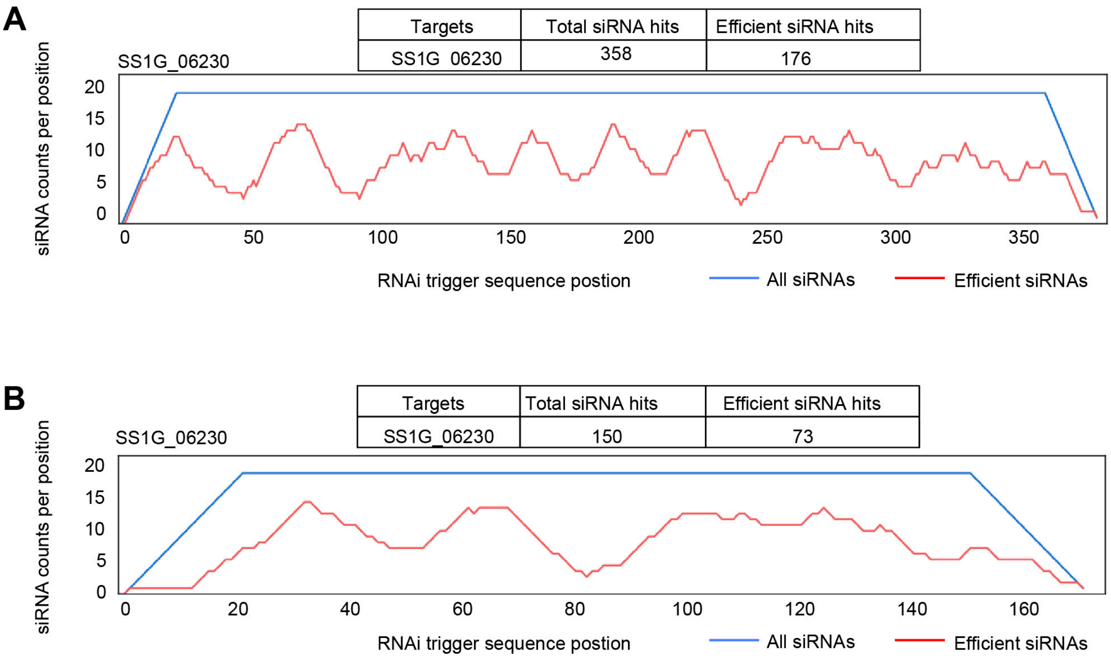

Figure S4. Off-target site prediction for the two SsYCP1 dsRNAs (SsYCP1A1 and SsYCP1A2)

No potential off-target site was predicted for SsYCP1A1 or SsYCP1A2. SsYCP1A1 and SsYCP1A2 can specifically target SsYCP1 (SS1G_06230) in *S. sclerotiorum*, and they have no target sites in tobacco.

Table S1. List of primers used in this study

Primer name	Sequence
SsYCP1qPCR-96F	GGCGCCGTTATCACTTTACTTG
SsYCP1qPCR-96R	ACCTGTGGTGTTTGATTGCG
SsYCP1(p35S)-Xba1F	TGGTCTAGAATGTTTCCCAAATTCGTTCTTC
SsYCP1(p35S)-Xma1R	AGACCCGGGTTAGGCACCGTTCGGCTGCCAAC
SsYCP1DSP(p35S)-Xba1F	TGGTCTAGAATGAAGACTCGGGGCCCATCTGATG
SsYCP1A1	TAATACGACTCACTATAGGGCACGCGGTGAGAACCATTC
SsYCP1B1	TAATACGACTCACTATAGGGTGTCGTTCCTACTAGAGTC
SsYCP1A2	TAATACGACTCACTATAGGGGGAAAATCATATTGGCACCAG
SsYCP1B2	TAATACGACTCACTATAGGGTCATGCGCAGCAATCACCACC

Table S2. All cupin domain-containing proteins in *S. sclerotiorum*

Sequence ID	Evaluate	Length (aa)	Family	Secreted
SS1G_06873	1.52E-133		Homogentisate dioxygenase	No
SS1G_01063	7.42E-125		Type I phosphomannose isomerase	No
SS1G_10796	3.09E-94	504	Germin/Seed storage 7S protein	Yes
SS1G_10384	3.04E-92		Type I phosphomannose isomerase	No
SS1G_08814	1.83E-91	445	Germin/Seed storage 7S protein	Yes
SS1G_12304	2.03E-84		Pirin-like	No
SS1G_04451	1.46E-58		3-hydroxyanthranilic acid dioxygenase-like	No
SS1G_06228	7.29E-50		YML079-like	No
SS1G_09378	1.84E-47		Pirin-like	No
SS1G_06230	2.67E-47	181	YML079-like	Yes
SS1G_05194	2.96E-47		YML079-like	No
SS1G_11285	1.41E-42		Cysteine dioxygenase type I	No
SS1G_00064	2.40E-41		Cysteine dioxygenase type I	No
SS1G_12493	5.34E-35		Ureidoglycolate hydrolase AllA	No
SS1G_11412	3.18E-27	370	Quercetin 2,3-dioxygenase-like	Yes
SS1G_00301	1.56E-26		Acireductone dioxygenase	No
SS1G_14354	1.04E-20		TM1287-like	No
SS1G_03393	4.69E-17		EutQ-like	No
SS1G_04766	1E-13	211	Germin/Seed storage 7S protein	Yes
SS1G_03843	2.53E-12		Hypothetical protein TM1112	No
SS1G_05201	5.35E-10		Germin/Seed storage 7S protein	No
SS1G_01635	2.17E-08		Gentisate 1,2-dioxygenase-like	No
SS1G_03550	1.13E-07		Germin/Seed storage 7S protein	No
SS1G_07561	3.77E-05		Hypothetical protein TM1112	No

SsYCP1 protein studied in this work is highlighted in green, while the other 4 cupins that have secretory signal peptides are highlighted in gray.

Table S3. Secretory signal peptide analysis of the 188 YML079-like cupin proteins in ascomycetes

ID	Strain	SP(Sec/SPI)	SP cleavage sit
XP_018378751	Alternaria alternata	Yes	18-19
XP_028501085	Alternaria arborescens	Yes	18-19
XP_028510761	Alternaria arborescens	No	
XP_024725068	Amorphotheca resinae ATCC 22711	No	
XP_038799156	Ascochyta rabiei	No	
XP_025507655	Aspergillus aculeatinus CBS 121060	No	
XP_020055124	Aspergillus aculeatus ATCC 16872	No	
XP_031904745	Aspergillus alliaceus	No	
XP_025446122	Aspergillus brunneoviolaceus CBS 621.78	No	
XP_031921110	Aspergillus caelatus	No	
XP_001259511	Aspergillus fischeri NRRL 181	No	
XP_041148144	Aspergillus flavus NRRL3357	No	
XP_753465	Aspergillus fumigatus Af293	No	
XP_025396938	Aspergillus heteromorphus CBS 117.55	No	
XP_025527830	Aspergillus japonicus CBS 114.51	No	
XP_041541743	Aspergillus luchuensis	No	
XP_026608205	Aspergillus mulundensis	No	
XP_681414	Aspergillus nidulans FGSC A4	No	
XP_001398744	Aspergillus niger CBS 513.88	No	
XP_015403700	Aspergillus nomiae NRRL 13137	No	
XP_024686063	Aspergillus novofumigatus IBT 16806	No	
XP_001824030	Aspergillus oryzae RIB40	No	
XP_031945295	Aspergillus pseudonomiae	No	
XP_031907875	Aspergillus pseudotamarii	No	
XP_041555044	Aspergillus puulaauensis	No	
XP_025468588	Aspergillus sclerotioniger CBS 115572	No	
XP_024702670	Aspergillus steynii IBT 23096	No	
XP_024710174	Aspergillus steynii IBT 23096	No	
XP_001212535	Aspergillus terreus NIH2624	No	
XP_026617606	Aspergillus thermomutatus	No	
XP_035351339	Aspergillus tubingensis	No	
XP_013428416	Aureobasidium namibiae CBS 147.97	No	
XP_029763385	Aureobasidium pullulans EXF-150	No	
XP_013340209	Aureobasidium subglaciale EXF-2481	No	
XP_013346973	Aureobasidium subglaciale EXF-2481	No	
XP_007673187	Baudoinia panamericana UAMH 10762	No	
XP_008595451	Beauveria bassiana ARSEF 2860	Yes	18-19
XP_008595453	Beauveria bassiana ARSEF 2860	No	
XP_014074016	Bipolaris maydis ATCC 48331	Yes	21-22
XP_014073061	Bipolaris maydis ATCC 48331	No	
XP_007683631	Bipolaris oryzae ATCC 44560	Yes	18-19
XP_007686967	Bipolaris oryzae ATCC 44560	No	

XP_007697528	Bipolaris sorokiniana ND90Pr	Yes	21-22
XP_007700307	Bipolaris sorokiniana ND90Pr	No	
XP_014558528	Bipolaris victoriae FI3	Yes	18-19
XP_014554694	Bipolaris victoriae FI3	No	
XP_007712095	Bipolaris zeicola 26-R-13	Yes	18-19
XP_007718104	Bipolaris zeicola 26-R-13	No	
XP_002626575	Blastomyces gilchristii SLH14081	Yes	28-29
XP_002625530	Blastomyces gilchristii SLH14081	No	
XP_001549406	Botrytis cinerea B05.10	No	
XP_037191998	Botrytis fragariae	No	
XP_041136575	Brettanomyces bruxellensis	No	
XP_028890310	Candida auris	No	
XP_025341485	Candida haemuloni	No	
XP_024715604	Candida pseudohaemulonii	No	
XP_007722998	Capronia coronata CBS 617.96	No	
XP_007734782	Capronia epimyces CBS 606.96	No	
XP_023459969	Cercospora beticola	No	
XP_016625753	Cladophialophora bantiana CBS 173.52	No	
XP_008731652	Cladophialophora carrionii CBS 160.54	No	
XP_016250185	Cladophialophora immunda	No	
XP_007739563	Cladophialophora psammophila CBS 110553	No	
XP_007761356	Cladophialophora yegresii CBS 114405	No	
XP_031892837	Colletotrichum fructicola	No	
XP_008094584	Colletotrichum graminicola M1.001	No	
XP_022470130	Colletotrichum orchidophilum	No	
XP_036589461	Colletotrichum truncatum	No	
XP_006674476	Cordyceps militaris CM01	Yes	19-20
XP_006674478	Cordyceps militaris CM01	No	
XP_020069613	Cyberlindnera jadinii NRRL Y-1542	No	
XP_008716047	Cyphellophora europaea CBS 101466	No	
XP_015466793	Debaryomyces fabryi	No	
XP_456971.2	Debaryomyces hansenii CBS767	No	
XP_020126616	Diplodia corticola	No	
XP_040659668	Drechmeria coniospora	No	
XP_007785529	Endocarpon pusillum Z07020	Yes	20-21
XP_013256949	Exophiala aquamarina CBS 119918	No	
XP_009160822	Exophiala dermatitidis NIH/UT8656	No	
XP_016224660	Exophiala mesophila	No	
XP_016258445	Exophiala oligosperma	No	
XP_016261196	Exophiala oligosperma	No	
XP_016231044	Exophiala spinifera	No	
XP_016241516	Exophiala spinifera	No	
XP_013311413	Exophiala xenobiotica	No	
XP_013320141	Exophiala xenobiotica	No	

XP_008024329	Exserohilum turcica Et28A	No	
XP_008026904	Exserohilum turcica Et28A	No	
XP_022516663	Fonsecaea monophora	No	
XP_016632443	Fonsecaea multimorphosa CBS 102226	No	
XP_022495230	Fonsecaea nubica	No	
XP_013284105	Fonsecaea pedrosoi CBS 271.37	No	
XP_031015912	Fusarium coffeatum	Yes	15-16
XP_031014628	Fusarium coffeatum	No	
XP_023436399	Fusarium fujikuroi IMI 58289	Yes	15-16
XP_023430550	Fusarium fujikuroi IMI 58289	No	
XP_011322188	Fusarium graminearum PH-1	Yes	15-16
XP_011319139	Fusarium graminearum PH-1	No	
XP_011323236	Fusarium graminearum PH-1	No	
XP_041681658	Fusarium mangiferae	No	
XP_031054247	Fusarium odoratissimum NRRL 54006	Yes	15-16
XP_031063946	Fusarium odoratissimum NRRL 54006	No	
XP_031033036	Fusarium oxysporum NRRL 32931	Yes	15-16
XP_031030907	Fusarium oxysporum NRRL 32931	No	
XP_031039449	Fusarium oxysporum NRRL 32931	No	
XP_031084662	Fusarium proliferatum ET1	Yes	15-16
XP_031083858	Fusarium proliferatum ET1	No	
XP_009263568	Fusarium pseudograminearum CS3096	Yes	21-22
XP_009255076	Fusarium pseudograminearum CS3096	No	
XP_009262485	Fusarium pseudograminearum CS3096	No	
XP_036541654	Fusarium subglutinans	No	
XP_037206339	Fusarium tjaetaba	No	
XP_003049213	Fusarium vanettenii 77-13-4	Yes	17-18
XP_003050813	Fusarium vanettenii 77-13-4	No	
XP_025585799	Fusarium venenatum	Yes	15-16
XP_025590836	Fusarium venenatum	No	
XP_018746764	Fusarium verticillioides 7600	No	
XP_009223485	Gaeumannomyces tritici R3-111a-1	Yes	23-24
XP_008085146	Glarea lozoyensis ATCC 20868	No	
XP_014169397	Grosmannia clavigera kw1407	No	
XP_001538872	Histoplasma capsulatum NAm1	Yes	30-31
XP_001542477	Histoplasma capsulatum NAm1	No	
XP_024733949	Hyaloscypha bicolor E	No	
XP_020073686	Hyphopichia burtonii NRRL Y-1933	No	
XP_003956104	Kazachstania africana CBS 2517	No	
XP_451734	Kluyveromyces lactis	No	
XP_022676362	Kluyveromyces marxianus DMKU3-1042	No	
XP_022459586	Kuraishia capsulata CBS 1993	No	
XP_022629782	Lachancea lanzarotensis	No	
XP_002553492	Lachancea thermotolerans CBS 6340	No	

XP_031008424	Lachnellula hyalina	No	
XP_003842489	Leptosphaeria maculans JN3	No	
XP_007295775	Marssonina brunnea f. sp. 'multigermtubi' MB_m1	No	
XP_007812168	Metarhizium acridum CQMa 102	Yes	22-23
XP_007812166	Metarhizium acridum CQMa 102	No	
XP_014543509	Metarhizium brunneum ARSEF 3297	Yes	22-23
XP_014543507	Metarhizium brunneum ARSEF 3297	No	
XP_007824968	Metarhizium robertsii ARSEF 23	Yes	22-23
XP_007824970	Metarhizium robertsii ARSEF 23	No	
XP_018065628	Mollisia scopiformis	No	
XP_003673564	Naumovozyma castellii CBS 4309	No	
XP_003669879	Naumovozyma dairenensis CBS 421	No	
XP_961056	Neurospora crassa OR74A	No	
XP_009848106	Neurospora tetrasperma FGSC 2508	No	
XP_013934977	Ogataea parapolymorpha DL-1	No	
XP_028487636	Paecilomyces variotii	No	
XP_001803273	Parastagonospora nodorum SN15	No	
XP_022493356	Penicillium arizonense	No	
XP_014530607	Penicillium digitatum Pd1	No	
XP_014533225	Penicillium digitatum Pd1	No	
XP_016595485	Penicillium expansum	No	
XP_040645966	Penicillium griseofulvum	No	
XP_002567737	Penicillium rubens Wisconsin 54-1255	No	
XP_007830356	Pestalotiopsis fici W106-1	Yes	23-24
XP_007829854	Pestalotiopsis fici W106-1	No	
XP_007911740	Phaeoacremonium minimum UCRPA7	No	
XP_030997906	Phialemoniopsis curvata	Yes	26-27
XP_001907504	Podospora anserina S mat+	Yes	21-22
XP_007919739	Pseudocercospora fijiensis CIRAD86	No	
XP_001937149	Pyrenophora tritici-repentis Pt-1C-BFP	No	
XP_030983172	Pyricularia grisea	No	
XP_023623430	Ramularia collo-cygni	No	
XP_013328864	Rasamsonia emersonii CBS 393.64	No	
XP_013270776	Rhinocladiella mackenziei CBS 650.93	No	
XP_016640590	Scedosporium apiospermum	Yes	23-24
XP_001593308	Sclerotinia sclerotiorum 1980 UF-70	Yes	19-20
XP_001593306	Sclerotinia sclerotiorum 1980 UF-70	No	
XP_001593766	Sclerotinia sclerotiorum 1980 UF-70	No	
XP_003349631	Sordaria macrospora k-hell	Yes	20-21
XP_016765915	Sphaerulina musiva SO2202	No	
XP_020116275	Talaromyces atroroseus	No	
XP_002144806	Talaromyces marneffei ATCC 18224	No	
XP_002340654	Talaromyces stipitatus ATCC 10500	No	
XP_004178963	Tetrapisispora blattae CBS 6284	No	

XP_003654547	Thermothielavioides terrestris NRRL 8126	Yes	24-25
XP_003654546	Thermothielavioides terrestris NRRL 8126	No	
XP_003680864	Torulaspora delbrueckii	No	
XP_024758636	Trichoderma asperellum CBS 433.97	No	
XP_013943288	Trichoderma atroviride IMI 206040	No	
XP_024753023	Trichoderma citrinoviride	No	
XP_018660556	Trichoderma gamsii	No	
XP_024777786	Trichoderma harzianum CBS 226.95	No	
XP_006966440	Trichoderma reesei QM6a	No	
XP_013960446	Trichoderma virens Gv29-8	No	
XP_011276816	Wickerhamomyces ciferrii	No	
XP_018191732	Xylona heveae TC161	No	
XP_002495585	Zygosaccharomyces rouxii	No	
XP_003857316	Zymoseptoria tritici IPO323	No	

Secretory signal peptide (SP) of effectors were predicted using SignalP-5.0 (http://www.cbs.dtu.dk/services/SignalP/)[3]. Sec/SPI: "standard" secretory signal peptides transported by the Sec translocon and cleaved by Signal Peptidase I (Lep).

References

1. Derbyshire, M.; Dentongiles, M.; Hegedus, D.; Seifbarghy, S.; Rollins, J.; Kan, J.V.; Seidl, M.F.; Faino, L.; Mbengue, M.; Navaud, O. The complete genome sequence of the phytopathogenic fungus *Sclerotinia sclerotiorum* reveals insights into the genome architecture of broad host range pathogens. *Genome Biology & Evolution* **2017**, *9*, 593-618.
2. Gough, J.; Karplus, K.; Hughey, R.; Chothia, C. Assignment of homology to genome sequences using a library of hidden Markov models that represent all proteins of known structure. *Journal of molecular biology* **2001**, *313*, 903-919.
3. Nielsen, H. Predicting secretory proteins with SignalP. In *Protein function prediction*; Springer: **2017**; pp. 59-73.
